# Investigating the antidiabetic efficacy of dairy-derived *Lacticaseibacillus paracasei* probiotic strains: modulating α-amylase and α-glucosidase enzyme functions

**DOI:** 10.3389/fmicb.2023.1288487

**Published:** 2023-12-04

**Authors:** Sujay S. Huligere, Chandana Kumari V B, Sudhanva M. Desai, Ling Shing Wong, Nagma Firdose, Ramith Ramu

**Affiliations:** ^1^Department of Biotechnology and Bioinformatics, JSS Academy of Higher Education and Research, Mysore, Karnataka, India; ^2^Department of Chemical Engineering, Dayanand Sagar College of Engineering, Bengaluru, Karnataka, India; ^3^Faculty of Health and Life Sciences, INTI International University, Nilai, Malaysia; ^4^Department of Pharmacology, JSS Medical College, JSS Academy of Higher Education and Research, Mysore, Karnataka, India

**Keywords:** diabetes mellitus, lactic acid bacteria, probiotics, α-amylase, α-glucosidase

## Abstract

The current study aims to evaluate and characterize the probiotic andantidiabetic properties of lactic acid bacteria (LAB) obtained from milk and other dairy-based products. The strains were tested physiologically, biochemically, and molecularly. Based on biochemical tests and 16S rRNA gene amplification and sequencing, all three isolates RAMULAB18, RAMULAB19, and RAMULAB53 were identified as *Lacticaseibacillus paracasei* with homology similarity of more than 98%. The inhibitory potential of each isolate against carbohydrate hydrolysis enzymes (α-amylase and α-glucosidase) was assessed using three different preparations of RAMULAB (RL) isolates: the supernatant (RL-CS), intact cells (RL-IC), and cell-free extraction (RL-CE). Additionally, the isolate was evaluated for its antioxidant activity against free radicals (DPPH and ABTS). The strain’s RL-CS, RL-CE, and RL-IC inhibited α-amylase (17.25 to 55.42%), α-glucosidase (15.08–59.55%), DPPH (56.42–87.45%), and ABTS (46.35–78.45%) enzymes differently. With the highest survival rate (>98%) toward tolerance to gastrointestinal conditions, hydrophobicity (>42.18%), aggregation (>74.21%), as well as attachment to an individual’s colorectal cancer cell line (HT-29) (>64.98%), human buccal and chicken crop epithelial cells, all three isolates exhibited extensive results. All three isolates exhibited high resistance toward antibiotics (methicillin, kanamycin, cefixime, and vancomycin), and other assays such as antibacterial, DNase, hemolytic, and gelatinase were performed for safety assessment. Results suggest that the LAB described are valuable candidates for their significant health benefits and that they can also be utilized as a beginning or bio-preservative tradition in the food, agriculture, and pharmaceutical sectors. The LAB isolates are excellent *in vitro* probiotic applicants and yet additional *in vivo* testing is required.

## Introduction

Diabetes mellitus (DM) is a medical condition described by inadequate secretion of insulin and/or diminished tissue responsiveness to insulin at any number of sites along the complex hormonal action pathways. Additionally, insulin resistance or irregularities in insulin secretion can be linked to other pathologies, such as excess thyroid hormone, glucocorticoids, growth hormones, or liver illness. These impairments coexist frequently and is difficult to determine the root cause responsible for hyperglycemia in the same patient (Poretsky, 2010). DM despite being a prevalent disease, its etiology is still unknown, most likely for a variety of ailments. The variation in type 2 diabetes caused by the interaction of numerous genetic and environmental variables, is possibly the most significant contributor. Roughly 100 million people or 4% of the global population, have type 2 diabetes mellitus (T2DM) (Kaul et al., 2012). In the United States of America, the level of incidence is greater, where it has affected 10.5% (approximately 34.2 million people) of the population and is surging at an incredible rate. More than 21% (approximately 7.3 million) of the adults in the USA affected by diabetes are undiagnosed, based on the data provided by the National Institutes of Health (NIH) and the Centers for Prevention and Control of Diseases (CDC) (Kaveeshwar and Cornwall, 2014; Diabetes Statistics – NIDDK, 2021). Inhibiting the enzymes α-amylase and α-glucosidase, which break down disaccharides and complex carbohydrates along the proximal gut brush border, causes carbohydrate absorption to be delayed and postprandial glucose rise to be lower (Fonseca and John-Kalarickal, 2010). Various pharmacological medications are accessible to assist with therapy intensification. Insulin stimulants (metformin and biguanide), insulin-secreting agents (sulfonylureas and non-sulfonylurea secretagogues), (GLP-1) agonists (including aldose reductase inhibitors, DPP4 inhibitors, and glucosidase inhibitors), and glucosidase inhibitors. Enzymes found in the small intestine brush border, such as α-glucosidase and α-amylase, are accountable for earning the breakdown of complex carbohydrates, whereas their inhibitors delay gastrointestinal (GI) intake of carbohydrates by avoiding their breakdown, with acarbose being one of the standard inhibitor drugs used. Ultimately, pramlintide, an equivalent of the peptide amylin, which the beta cell co-secretes with the hormone insulin, is indicated to be used in patients with both type 1 and type 2 diabetes in conjunction with insulin (Alam et al., 2014).

Continuous administration of these pharmacological drugs as treatment causes renal impairment, cardiovascular illness, diminished appetite, fluid retention, and recurrent GI tract infection, among other negative effects. In this context, gut microbiota plays a significant part in sustaining certain diabetic metabolism (Martiz et al., 2023). The enhancement of the host’s health depends on changing the microbes in their gut to achieve or maintain a favorable condition (Sreepathi et al., 2022). In comparison to other commonly available drugs, utilizing it for treatment offers less known adverse effects. Administration of these live microorganisms, which are commonly termed probiotics – aids in altering the GI ecology (Kumari et al., 2022a,b). Probiotics have been proven to decrease the growth and adherence of potentially dangerous microbes (Kumari et al., 2022a,b). There is substantial evidence that probiotic bacteria may communicate with lymphoid tissue associated with the gut and alter the levels of local oral immunity and immune system function (Qin et al., 2005).

LABs are well-known bacterial species that were recently discovered as being suitable probiotics (GRAS). In addition, when taken, lactic acid-producing bacteria like a type of probiotic provide several health benefits to the host (Sreepathi et al., 2023), including antibacterial, antioxidant, and anti-diabetic capabilities. LAB also assists in the fermentation process for a variety of sources; numerous studies have demonstrated that LAB fermentation of food increases the volume, availability, digestibility, and assimilation of nutrients (Widyastuti et al., 2014). Despite claims of health and nutritional benefits for LAB in fermented dairy products dating back almost a century, the nutritional and therapeutic utility of these organisms is still controversial (Gemechu, 2015). Dairy products or sources are the most common source of LAB. The process of boiling milk is claimed to be a successful way of controlling disease-causing microbes. Furthermore, bacteria such as *Salmonella, Listeria*, and Q fever bacteria are frequently found in raw milk (Pexara et al., 2018). Existing pathogens are eliminated by heating the milk adequately, pasteurization, ultra-heat treatment, or boiling. Hence, consuming boiled milk or products made after pasteurization is always advised. Acidification occurs through bacterial fermentation or the incorporation of an acid, like lemon juice or vinegar, that lowers the pH. The acid causes milk proteins (mostly casein) to coagulate and thicken, restricting the growth of dangerous germs and prolonging the lifespan of the item. Earlier studies have indicated that LAB isolated from dairy sources has exhibited positive results in aiding various health benefits (Ağagündüz et al., 2021). The consumption of probiotic LAB in dietary supplements as well as the intake of dairy products and fermented foods constitutes one of the safest and newest techniques for biotherapy which results in health benefits (Patil et al., 2023).

Yet, the processes beneath the positive effects of probiotic biomedical treatment remain insufficiently understood, and the research that’s currently accessible is either unfinished or circumstantial (Harun-ur-Rashid et al., 2007). Considering this context, the current research aims to evaluate the anti-diabetic benefits of probiotic LAB species isolated from dairy products by inhibiting the glycogen-hydrating enzymes α-glucosidase and α-amylase. In the present research, the LAB strains are isolated from dairy sources, namely from raw milk and overnight fermented curd prepared from boiled milk by acidification. The current research also assesses various traits of the identified LAB strains from dairy sources, including tolerance, safety, exopolysaccharide production, molecular identification, phylogenetic analysis, and cell adherence capabilities. After *in vivo* evaluation studies, the isolated LAB strains from the study can be formulated into a biopharmaceutical drug that can be utilized as an alternative to treat diabetes if the study delivers the required results.

## Materials and methods

### Materials

The compounds employed in this investigation were generously supplied by HiMedia Laboratories Pvt. Ltd., situated in Mumbai, India. These included MRS (de Man, Rogosa, and Sharpe; agar and broth), NaCl, ox gall salt, phenol, xylene, deoxyribonuclease (DNase) agar medium, Blood agar medium supplemented with 5% (w/v) sheep blood, ABTS [2,2′-azino-bis (3-ethylbenzothiazoline-6-sulfonic acid)] and DPPH (2,2-diphenyl-1-picrylhydrazyl), as well as antibiotic susceptibility discs. The microbial strains subjected to analysis were procured from the Microbial Type Culture Collection and Gene Bank (MTCC) located in Chandigarh.

### Bacterial isolation

A lactating cow’s milk was collected early in the morning and brought to the laboratory under cold conditions from a local cow farm situated in Nanjangud, located at coordinates 12° 7′ 12.0000″ N and 76° 40′ 48.0000″ E, within the state of Karnataka, India. A portion of the same milk was later used for making curd. The milk was boiled and cooled, and a spoonful of lemon juice was added to the milk and kept aside for overnight curd settling. The overnight settled curd and milk were both used as samples for LAB isolation (Perea Vélez et al., 2007). The samples were diluted with saline several times before being pour-plated onto an MRS agar plate under an anaerobic condition at around 37°C for 24–48 h. Fresh cultures from colonies with a variety of physical features have been smeared onto the MRS agar plate. The fully grown cultures were subsequently maintained in MRS broth with 40% v/v glycerol at −40°C.

### RL-CS, RL-IC, and RL-CE preparation 1×10^8^ CFU/mL

To summarize, all three RAMULAB isolates were cultured in MRS broth for 24 h under anaerobic circumstances at 37°C. The overnight cultures were adjusted to a concentration of 1×10^8^ CFU/mL, and the supernatant was collected by centrifuging at 10,000 rpm for 15 min at 4°C. The residual bacteria and debris were removed from the resultant supernatant using a 0.22 μm membrane filter before it was used as RL-CS (Cell-free supernatant). To collect RL-IC (Intact Cells), overnight, cultures were centrifuged at 3,000 rpm for 10 min at 4°C. The recovered pellets were further adjusted to 1×10^8^ CFU/mL (Calibration: adjusting the concentration to 0.5 McFarland standards turbidity by measuring its optical density at 625 nm) and used as RL-IC after being rinsed with PBS (pH 7.4). After centrifugation at 6000 rpm for 10 min at 4°C, overnight, cultures were recovered and washed with PBS. Cells have been sonicated for 25 min at 4°C using glass beads (0.3 mm dia, 50 rpm) in 50 mM Tris hydrochloride buffer (pH 7.0). After the homogenization process, glass beads and cellular debris were eliminated through centrifugation at 15,000 rpm for 15 min at a temperature of 4°C. Subsequently, centrifugation was performed to eliminate any remaining insoluble components. The resulting transparent supernatant was designated as the cell-free extract (RL-CE).

### Initial biochemical analysis

Biochemical analyses represent the classic phenotypic attributes of lactic acid bacteria (LAB) encompass morphological aspects (cellular and colony features such as shape, color, and texture), physiological factors (growth at diverse temperatures: 4°C, 10°C, 37°C, 45°C, and 50°C; tolerance to varying salt concentrations: 2, 4, 8, and 10%; and pH tolerance: 2, 4, 6, and 8), as well as biochemical characteristics. The characterization of the isolates used in this study followed the guidelines outlined in Bergey’s Manual of Determinative Bacteriology (Cowan, 1948).

### Probiotic evaluation

#### Tolerance assays: bile acid and simulated digestive conditions and phenol tolerance

The LAB isolates have been assessed for acid tolerance and ox gall salt (0.3 and 1% concentration) to determine their percentage of survivability; the methodology was completed as described by Chou et al. MRS broth with 0.3 and 1% concentration ox gall salt was prepared and the pH was adjusted to 2 (100 μL) inoculated LAB was incubated at 37°C. Cells were counted at 2 h intervals following inoculation (Chou and Weimer, 1999). The gastrointestinal stimulation and intestinal juice were made by immersing pepsin (3 × 10^6^ μg/L) and trypsin (1 × 10^6^ μg/L), (SRL Pvt. Ltd., Mumbai, India) in PBS at pH 2 and pH 8, respectively, and then sterilizing them with a 0.22 μm filter membrane. The methodology was carried out as previously stated by Mazahreh and Ershidat (2009). The cultivated isolates (1×10^8^ CFU/mL; 1,000 μL) were injected into simulated digestive gastric juice (9 mL) and incubated for different intervals (0,1 and 3 h; 5% CO_2_ incubator; 37°C). Following incubation, the cells were placed into simulated digestive intestinal juice (9 mL) and incubated at different intervals (0,1,3 and 8 h; 5% CO_2_ incubator; 37°C). Following successive dilution steps and an incubation period at 37°C lasting 24 h, the viable colony count of the isolated LAB strains was determined using the spread plate technique. The formula to calculate the survival percentage is as follows:


Survival(%)=[(log)CFUL1/(log)CFUL0]×100

Where: CFU L1 is the number of LAB strains after treatment. CFU L0 is the number of LAB strains before treatment.

Jena et al. created a system for determining the phenolic rate of survival of cells from LAB isolates. In brief, LAB (1× 10^8^ CFU/mL) was cultured in MRS broth containing 0.4% phenol for 24 h. The culture was plated on MRS agar for an overnight duration, and viable cells were identified using the colony count technique (Klongklaew et al., 2022).

#### Exopolysaccharide production

The test for exopolysaccharide (EPS) production was carried out using a milk-ruthenium medium following Abushelaibi et al. (2017) instructions. Overnight LAB (1 × 10^8^ CFU/mL) cultures were plated on ruthenium red milk media plates (10% (w/v) skim milk powder, 1% (w/v) sucrose, 80 mg/L ruthenium red, and 1.5% (w/v) agar). The appearance of a white bacterial cell wall of the colonies after 48 h of incubation at 37°C, confirms the production of EPS (Abid et al., 2018).

### Cell adherence assays

#### Cell surface hydrophobicity

The cell’s hydrophobicity of the surface in the LAB isolates has been examined against xylene, a non-polar hydrocarbon. The cell surface’s hydrophobic properties were determined by observing a decrease in aqueous phase absorbance at 600 nm. The method previously laid out by Li et al. was employed to look into the cell contact with the surface (Qing et al., 2015). The following assay was carried out and determined utilizing the equation provided below:


$$\mathrm{Hydrophobicity}\;\left(\%\right)=\left[1–\left(H/H_{0}\right)\right]\times 100.$$

Where: H represents the ultimate absorbance of the aqueous phase, while H_0_ stands for the initial absorbance.

#### Autoaggregation

During time points of 0, 2, 4, 6, 10, and 24 h, the autoaggregation % of the LAB strains was determined utilizing the method described by Taleb et al. The autoaggregation of the isolates was performed using 18 h of cultured cells that were reconstituted in PBS (1 × 10^8^ CFU/mL) (Tareb et al., 2013). This equation was used to compute the Autoaggregation %.


$$\mathrm{Autoaggregation}\;\left(\%\right)=\left[1–\left(X_{t/}X_{0}\right)\right]\times 100$$

Where X_t_ and X_0_ indicate the absorbance at the time “t” and at the initial time “0,” respectively.

#### Coaggregation

Coaggregation between the RAMULAB isolates and opportunistic pathogens (*Micrococcus luteus* MTCC:1809, *Escherichia coli* MTCC:4430, *Pseudomonas aeruginosa* MTCC:424, *Salmonella typhimurium* MTCC 98, and *Bacillus subtilis* MTCC 10403) was assessed at 37°C for 4 h of incubation as described by the method by Ekmekci et al. (2009).

$\mathrm{Coaggregation}\%=\left[\left(X_{L}+X_{P}\right)–X_{\mathrm{mix}}/\left(X_{L}+X_{P}\right)\times 100\right]$

Where Xmix signifies [the absorbance of the RAMULAB mixture + pathogen at 4 h], and X_L_ + X_P_ denotes [the absorbance of the RAMULAB mixture + pathogen at time 0 h].

#### Adherence to chicken crop and human buccal epithelial cells

LAB adhesion to epithelial cells of chicken crop was investigated as per the previous method by Somashekaraiah et al. LAB isolates were mixed in a 1:10 ratio with epithelial cells of the chicken crop (1 × 10^6^ cells/mL), followed by incubation for 1 h at the ideal temperature (37°C). After incubation, the non-adherent bacterial cells were separated by centrifugation (3,000 rpm, 5 min). The pellets were washed and resuspended in 100 μL of phosphate saline and viewed under the microscope after crystal violet staining (Somashekaraiah et al., 2019). The procedure utilized in the prior investigation by Kumari et al. (2022a,b) was applied to examine the LAB isolate potential to bind human buccal epithelial cells in an *in vitro* condition.

#### Adherence to HT29 cells

The human adenocarcinoma of the colorectal tumor cell line HT-29 was utilized to investigate LAB adhesion to intestinal cells, exactly as described by Fonseca et al. (2021). To assess their ability to adhere, HT-29 cells were placed at a density of 10^5^ cells/well on 6-well tissue culture plates and cultured at 37°C; for 24 h (5% CO_2_ and 95% air environment). The separated cells from 18-h cultures were resuspended in non-supplemented DMEM media while being rinsed twice using Dulbecco’s phosphate-buffered saline (DPBS). The HT-29 cell monolayers seeded earlier are then supplemented with these bacterial suspensions. During 2 h of culture at 37°C under a 5% CO_2_ and 95% air environment, every well was meticulously rinsed a total of three times using PBS to get rid of the suspensions of bacteria and ineffective cells. A 1% solution of Triton X-100 (HiMedia, India) was employed to remove the adherent microorganisms. On MRS agar, the final viable LAB cell counts were counted as Log CFU/mL (Fonseca and John-Kalarickal, 2010). The adherence rate of the strains to LAB was obtained utilizing the equation below:


$$\mathrm{Adhesion rate}\;\left(\%\right)=\left(X/X_{0}\right)\times 100$$

Where X = Number of adherent cells, X_0_ = Initial number of cells inoculated.

### Safety assessment

#### Antibiotic sensitivity

The disc diffusion method was used to determine antibiotic susceptibility for LAB isolates (10^8^ CFU/mL) in compliance with the Clinical and Laboratory Criteria Institutes (CLSI) 2018 criteria. In the context of assessing antibiotic susceptibility, a series of antibiotic discs were utilized, each corresponding to a specific antibiotic. These included: Clindamycin (CL, 2 mcg/disc), Chloramphenicol (C, 30 mcg/disc), Ampicillin (AMP, 10 mcg/disc), Gentamicin (GEN, 10 mcg/disc), Tetracycline (TET, 30 mcg/disc), Kanamycin (KAN, 30 mcg/disc), Rifampicin (RIF, 5 mcg/disc), Vancomycin (VAN, 30 mcg/disc), Methicillin (MET, 5 mcg/disc), Erythromycin (ERY, 15 mcg/disc), Streptomycin (STR, 100 mcg/disc), Cefixime (CFX, 5 mcg/disc), and Azithromycin (AZM, 15 mcg/disc). The outcomes resulting from this evaluation were classified into three categories: Resistant (R), Susceptible (S), and Moderately Susceptible (MS). These categorizations were determined based on the diameter of the zone of inhibition observed in the testing. The assessment process adhered to the Performance Standards for Antimicrobial Disc Susceptibility Tests, as outlined by the Clinical and Laboratory Standards Institute (CLSI) scale (Temmerman et al., 2003).

#### Hemolytic activity

The investigation was conducted using blood agar plates (HiMedia, Mumbai, India) to assess the hemolytic activity of the LAB isolates, following the methodology outlined by Somashekaraiah et al. Hemolytic activity was gauged by quantifying the extent of red blood cell lysis in the vicinity of each colony. The presence of a transparent area encircling the colonies indicated a distinct zone of safety (Somashekaraiah et al., 2019).

#### DNase activity

Subsequently, the LAB isolates were streaked onto a deoxyribonuclease (DNase) agar medium to assess their potential for generating DNase enzymes. Following a 48 h incubation period at 37°C, indications of positive DNase activity were discovered in a distinct zone around the colonies. The experiment was carried out exactly as stated by Abouloifa et al. (2020).

#### Antimicrobial activity

As per Barzegar et al. methodology with a few minor improvements, pathogenic strains and the antibacterial activity of RAMULAB isolates [*Bacillus cereus* MTCC:1272, *Staphylococcus aureus* MTCC:1144, *S. typhimurium* MTCC:98, *E. coli* MTCC:443, *P. aeruginosa* MTCC:424, *Klebsiella pneumoniae*, *M. luteus* MTCC:1809, *B. subtilis* MTCC:10403, *Pseudomonas florescens* MTCC:667 and, *Klebsiella aerogenes* (*E. aerogenes*) MTCC:2822 was evaluated]. Briefly, 50 μL overnight cultured indicator pathogen was overlaid onto the Luria Bertani agar (LB agar) plates. For the agar well method, on the hardened agar, wells were formed. 100 μL of 18-h overnight grown LAB isolates were inoculated into the well, let to dry, and then incubated at 37°C for 24–48 h (Kumar et al., 2022). The diameter of the well’s lateral zone of inhibition was measured, and positive inhibition was defined as a clear zone of 1 mm or greater (Barzegar et al., 2021).

#### Molecular identification and phylogeny

The target isolates have been determined utilizing molecular methods involving 16s ribosomal-RNA sequencing using the PCR conditions outlined by Endres et al. (2021). Amplification was carried out using the universal bacterial forward primer-27F and reverse primer-1492R. MEGA X (Version 11) was used to generate a phylogenetic tree from the 16s rRNA region of the three LABs isolated in this work. The phylogenetic tree with the highest likelihood is built using 1,000 bootstrap consensus trees. The Tamura-Nei model was chosen because it best fits (Tamura et al., 2007). The Neighbor-Join and BioNJ algorithms were used to automatically create the initial tree(s) for the systematic search on a matrix of pairwise distances (Agaliya and Jeevaratnam, 2013).

### Inhibitory activities

#### Antioxidant activities

LAB isolates were tested for their ability to scavenge DPPH ABTS radicals at 10^3^, 10^6^, and 10^9^ CFU/mL cell concentrations using the approach published by Jeong et al. (2021). For the DPPH experiment, 20 μL of the LAB culture was combined with 50 μL of DPPH (0.04 mg/mL) and incubated at room temperature for 30 min under dark circumstances before measuring absorbance at 517 nm. Alternatively, to perform the ABTS assay, 50 μL of the LAB culture was mixed with 950 μL of ABTS (0.07 mg/mL). The absorbance at 734 nm was measured shortly after 12 h of incubation in the dark. The following equation was used to calculate the radical scavenging activity:


$$\mathrm{Scavenging rate}\;\left(\%\right)=\left[1–\left(X_{s}/X_{b}\right)\right]\times 100$$

where X_S_ = absorbance of the reactants with the sample and X_b_ = absorbance of the reactants without the sample.

#### Carbohydrate hydrolyzing enzyme activities

To carry out the α-glucosidase and α-amylase inhibition activities, minor modifications were made to the methods detailed by Kwun et al. and Ramu et al. respectively. For the α-glucosidase inhibition assay – 700 μL of PBS (50 mM, pH 6.8) was mixed with RL-CS, RL-CE, and RL-IC of the RAMULAB isolates and incubated for 10 min. This combination was pre-incubated for 15 min at 37°C with the enzyme α-glucosidase (100 μL, 0.25 U/mL). As a substrate, 100 μL of p-nitrophenyl-D-glucopyranoside (pNPG, 5 mM) has been added to this mixture and incubated for 30 min at 37°C. The enzymatic process was halted by adding 1 mL of Na_2_CO_3_ (0.1 M). For the α-glucosidase inhibition experiment, the absorbance was measured at 405 nm. Following that, 500 μL RL-CS, RL-CE, and RL-IC of the LAB isolates were pre-incubated for 10 min at 25°C in 500 μL of PBS (0.1 M, pH 7.4) containing α-amylase enzyme (0.5 mg/mL) 0.500 μL solution containing 1% starch after a 10 min incubation at 25°C, 1 mL of 3, 5-dinitro salicylic acid reagents were added to a hot water bath that was boiling for 5 min to cease the enzyme process until cooling. After adding and diluting the mixture with 10 mL of distilled water, the absorbance at 540 nm was measured. The inhibitory effect in RAMULAB isolates was calculated using the equation below (Chen et al., 2014; Ramu et al., 2014; Kwun et al., 2020).


$$\mathrm{Inhibition}\;\left(\%\right)=\left[1–\left(X_{s}/X_{c}\right)\right]\times 100$$

Where Xc denotes the rate of absorption of the reactants in the absence of the sample and XS denotes the rate of absorption of the reactants when paired with the material being studied.

#### Statistical analysis

All experiments were done in triplicate. On graphs, the standard deviation is displayed as error bars (GraphPad Prism Software Inc., San Diego, CA, USA). ANOVA was used to evaluate the data. The significance of the differences was determined using the *p* ≤ 0.05 test.

## Results and discussion

Dairy-fermented products and milk are the cornerstones of global dietary lifestyles. Fermented dairy products have long been known to provide health advantages beyond their nutritional and organoleptic qualities (García-Hernández et al., 2016). Dairy products, especially those that have been fermented, are an important source of LAB (Kim and Lim, 2019). These microorganisms have a variety of beneficial effects on their host, including the production of bioactive substances during the process of fermentation, interactions of living cells with naturally occurring microbiota in the gastrointestinal environment, and the release and/or induction of signals that can control complex physiological communications (Hardy et al., 2013). Despite a lack of conclusive data, studies on the association between dairy products consumed and diabetes prevalence reveal an inverse relationship. Diabetes mellitus is a chronic illness characterized by hyperglycemia caused by resistance to insulin or lack of insulin release from pancreatic β-cells (Everard and Cani, 2013). Dairy products are not analyzed following their subgroups, which accounts for the inconsistent results (fatty, fat-free, fermented, etc.). In prior research, it has been found that dairy products, particularly low-fat and fermented milk products, help to lower the prevalence of diabetes. These outcomes are made possible by dairy LAB strains that exhibit probiotic qualities and produce bioactive peptides, which reduce oxidative stress, control gut flora, and have immune-modulating, antioxidative, antihyperglycemic, and anti-inflammatory effects (Colombo et al., 2018). The current work is looking into methods to suppress both carbohydrate-hydrolyzing enzymes α-amylase and α-glucosidase using probiotic *Lacticaseibacillus paracasei* strains derived from dairy products.

### Preliminary characterization

The biochemical characteristics of a bacterium provide multiple features useful for classification and recognition. The most popular and cost-effective method for establishing the genus and species of bacteria is to analyze the nutritional and metabolic characteristics of the bacterial isolate (Kumari et al., 2023). A combined total of 15 isolates from curd and milk samples were first tested for LAB. Based on their phenotypic features, only three of the potent isolates (two from milk and one from curd) were classified as LAB. By morphology, all three isolates were rods (bacilli), Gram-positive and catalase-negative. According to biochemical analysis, the isolates were hetero-fermentative, with no gas emission during glucose fermentation. All three strains grew normally at 37°C, although strain RAMULAB53 could resist temperatures as high as 45°C. Only at 2 and 4% salt concentrations in the media did optimal development occur. The isolates thrived significantly at pH 2, 4, and 6, but best at pH 7.4. The lactose glucose, maltose, sucrose, galactose, and mannitol could all be fermented by all three isolates (Table 1).

**Table 1:** The phenotypic, biochemical, and fermentation capacity of LAB strains isolated from curd and milk samples.

	Sample	Milk	Milk	Curd
	Isolates	RAMULAB18	RAMULAB19	RAMULAB53
Tests	Gram staining	Positive	Positive	Positive
	Catalase	Negative	Negative	Negative
	Morphology	Short-Rod	Rod	Short Rod
	Indole	Absence	Absence	Absence
	Methyl Red	Presence	Presence	Presence
	Voges Proskauer	Absence	Absence	Absence
	Citrate	Absence	Absence	Absence
	Starch hydrolysis	Absence	Absence	Absence
	Gelatin liquefaction	Absence	Absence	Absence
Temperature-related growth (°C)	4	Absence	Absence	Absence
	10	Absence	Absence	Absence
	37	Presence	Presence	Presence
	45	Absence	Absence	Presence
	50	Absence	Absence	Absence
Salt–related growth (%)	2	Presence	Presence	Presence
	4	Presence	Presence	Presence
	8	Absence	Absence	Presence
	10	Absence	Absence	Absence
Carbohydrates fermentation	Lactose	Presence	Presence	Presence
	Glucose	Presence	Presence	Presence
	Maltose	Presence	Presence	Presence
	Sucrose	Presence	Presence	Presence
	Mannitol	Presence	Presence	Presence
	D-xylose	Absence	Absence	Presence
	L-xylose	Absence	Absence	Absence
	Galactose	Presence	Presence	Presence
	Arabinose	Absence	Absence	Absence
	Starch	Absence	Absence	Absence

### Evaluation of probiotic attributes

#### Tolerance assays

##### Bile acid and simulated digestive conditions

**Figure 1:**
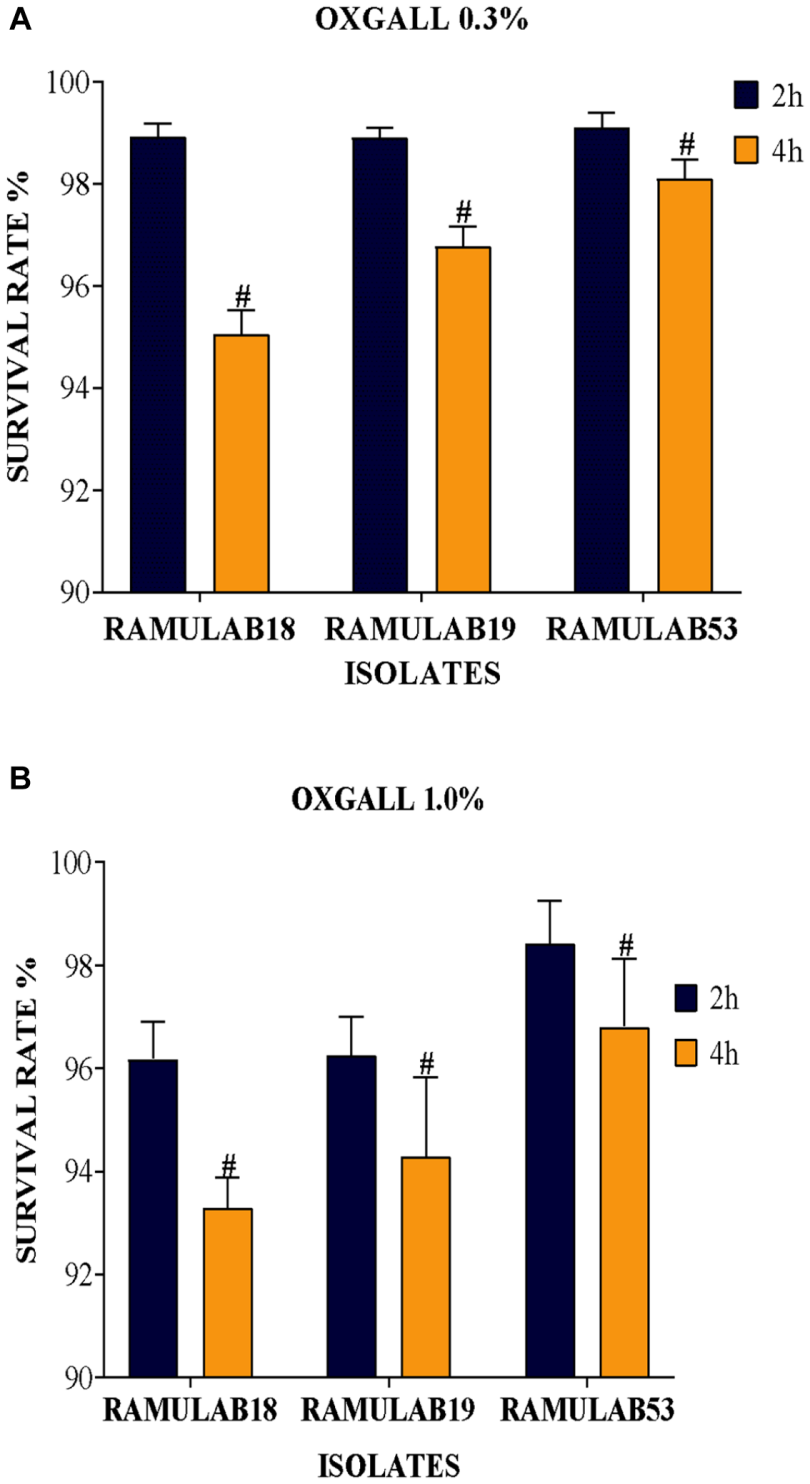
**(A)** Oxgall 0.3% **(B)** Oxgall 1% depicts the survival of isolates on MRS agar plates at 37°C for 2 and 4 h under acidic pH 2 conditions and different bile salt concentrations, with the data presented as a mean standard deviation; the application of Duncan’s range analyses revealed substantial variability among average survival rates, marked with superscript “#” to signify significant differences (*p* ≤ 0.05).

One of the important qualities allowing bacteria with lactic acid to survive in the small intestinal tract is biliary resistance to salt in very acidic circumstances. Arqués et al. report that three *L. rhamnosus* strains, type strain ATCC 7469, commercialization strain *L.* GG, and the strain having a superior competence to develop in the presence of bile salts, were studied on stress caused by acidic incubation. The stress was visible after the first hour of incubation, but the serious injury showed after 4 h in some cases and appeared to be closely linked to the incubation pH, medium, and acids (Arqués et al., 2015). Elbanna et al. discovered that the strain *B. velezensis* R7-1003 was resistant to low pH (2) and higher bile salt concentration (0.3%), as well as having greater survival capability after high-temperature exposure (60, 70, and 80°C)(Elbanna et al., 2018). In comparison to a previous study, the acid bile tolerance in our study supports the LAB isolates’ survival rate at high pH 2 as well as bile salt tolerance (0.3 and 1%), as shown in Figure 1. Figure 1 depicts the LAB isolates’ potential for longevity at pH 2 as well as their sensitivity to 0.3 and 1% of bile (a, and b), respectively. When analyzed alongside the remaining two isolates, the LAB isolate RAMULAB18 had the lowest inhibition rates of 95.05 and 93.82% after 4 h of incubation for 0.3 and 1% acid bile, respectively. At 0.3% bile content, the isolates RAMULAB19 and RAMULAB53 had a high survival rate of 98.90 and 99.10%, respectively.

##### Simulated gastrointestinal juice tolerance assay

**Figure 2:**
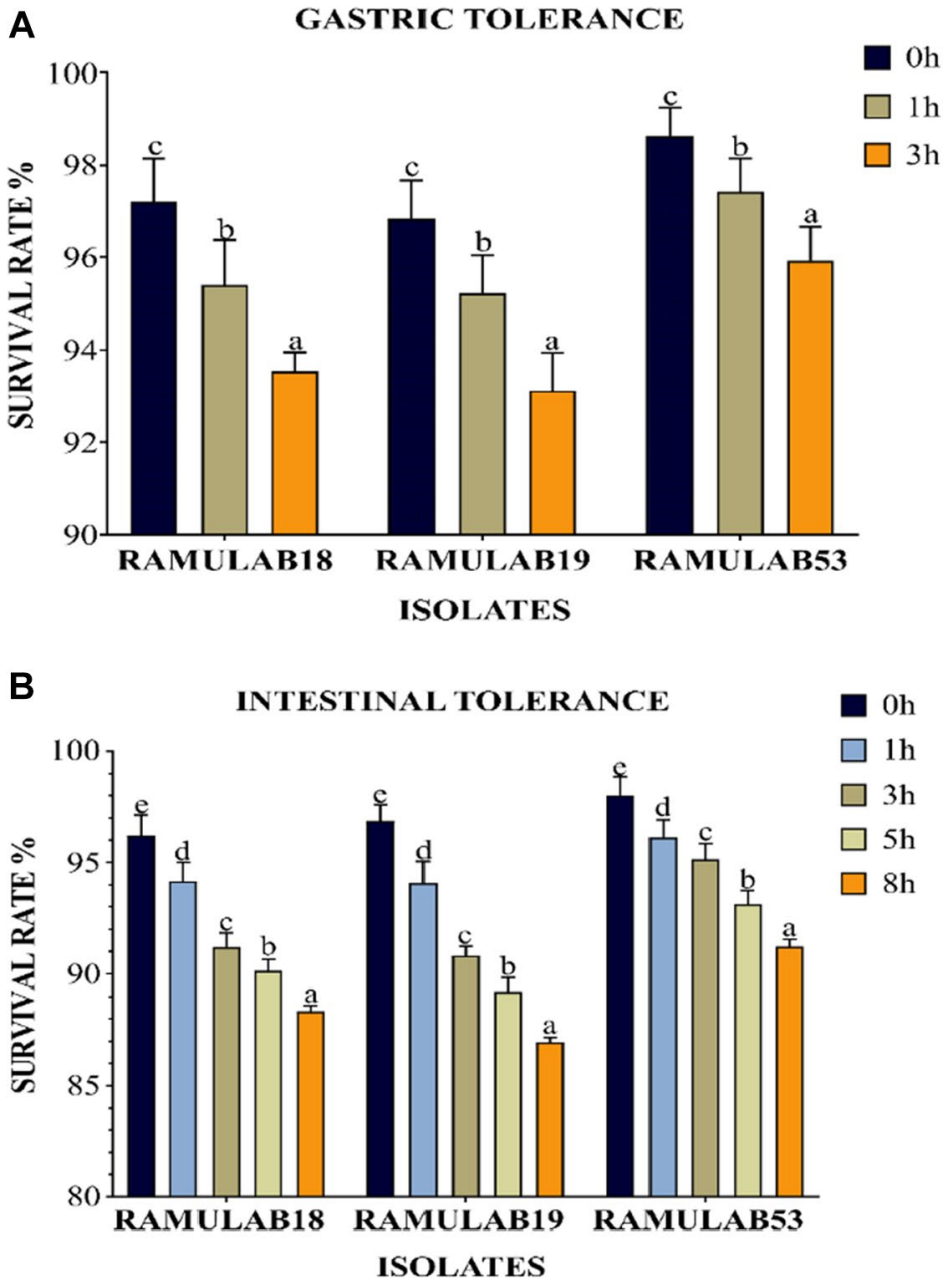
The survival rate (%), **(A)** gastric and **(B)** intestinal juice tolerance of LAB strains adhering to 37°C incubation throughout various survival time intervals is shown. Mean standard deviation was employed to represent the data, and the utilization of Duncan’s multiple range tests indicated noteworthy distinctions among survival rates, denoted by superscripts (a–e), alluding to statistically significant differences (*p* ≤ 0.05).

To be effective as a probiotic, LAB must be able to withstand the acidic environment of the stomach and the intestinal environment at pH 2 and 8, respectively, as well as resist bile acid concentrations and have an antagonistic effect on pathogenic organisms (Fooks et al., 1999). One of the selection criteria for probiotic microorganisms is resistance to low pH. These microorganisms must navigate through the complex environment of the stomach (pH 2, 3 h) to reach the small intestine (pH 8, 8 h). Although pH can drop as low as 1 in the stomach, pH 2 has traditionally been recommended by *in vitro* tests (De Filippis et al., 2020). Due to the frequent observation that strain viability declines significantly at pH 2.0 and lower. The viability of the three isolates was assessed with a gastrointestinal assay that mimics the process of food digestion under pH 2 for stomach conditions and pH 8 for intestinal conditions over a progressive period of 3 to 8 h, respectively. The three isolates were able to remain alive at the two pHs throughout the incubation period. All three *L. paracasei* strains were consistently resistant to low pH. This was comparable to an Argyri et al. In a study, nine *Lactobacillus* strains (*L. plantarum*, *L. pentosus*, *L. paracasei* subsp. paracasei) with final populations of more than 8 Log CFU/mL showed great tolerance of lower pH (Argyri et al., 2013). Compared to prior research, ours had a higher likelihood of survival and tolerance for digestive acids and gastroenteritis. All three isolates grew optimally in both stomach and intestinal environments, with a considerable survival rate between 3 and 8 h. Upon time, a decrease in the survival rate was observed in all three isolates. At 3 and 8 h, the RAMULAB53 isolate had the best survival rate, ranging from 95.92 to 91.21% for gastrointestinal and intestine diseases, respectively. Under gastrointestinal conditions, all three isolates demonstrated a considerable survival rate (Figures 2A,B).

##### Resistance to phenol and exopolysaccharide production

The phenolic conditions produced by microbiological destruction of amino acids that are extracted from dietary proteins can aid in the survival of the gut microbiota. Gut microorganisms can produce phenol and other dangerous compounds that are released during digestion. Therefore, any bacteria that can survive in these circumstances can be regarded as having probiotic potential (Arqués et al., 2015). Elbanna et al. found that at 0.2 and 0.4% phenol concentrations, the long-term survival rates of the strains Pro 4 & Pro 7 as 98, 98, 80, and 72%, respectively. Up to a concentration of 0.4%, both isolates showed good phenol resistance; however, as the phenol concentration increased, the survival rate rapidly dropped. Similarly in our investigation, after 24 h of incubation, the cell viability dropped from 7.09 to 6.94 log CFU/mL with a 0.4% phenol, suggesting LAB cell viability with phenol resistance in the GI tract. It was found in this investigation that the isolates were very good at tolerating phenol and survived along the GI tract. The current research investigates the incubation of LAB isolates for 0 and 24 h with 0.4% phenol. All the isolates expressed equivalent growth. The number of viable cells ranged between 6.94 and 7.32 Log CFU/mL. The isolated RAMULAB53 demonstrated the greatest tolerance among the other strains, measuring 7.32 Log CFU/mL.

**Figure 3:**
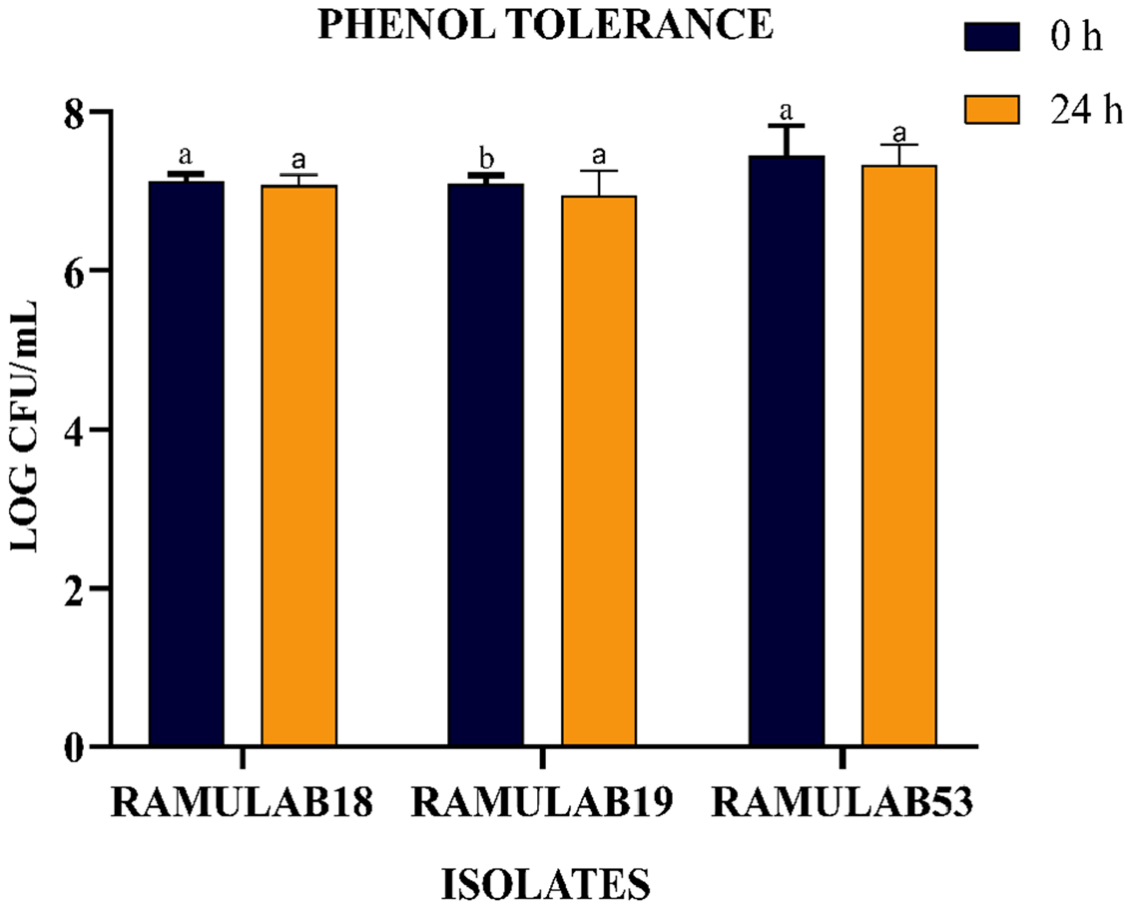
LAB strain phenol tolerance after 0 and 24 h. The data shows mean standard deviation, and Duncan’s tests confirmed significant differences (*p* ≤ 0.05) in survival rates and superscripts (a,b).

Dairy products that have been fermented may be beneficial to one’s health due to the effects of microbial byproducts (biogenic or bioactive effects) created through the process of fermentation, in addition to the probiotic benefits provided by specific LAB strains separated by their content (Elbanna et al., 2018). Peptides, exopolysaccharides (EPSs), bacteriocins, numerous amylases, protease, and lipase enzymes, as well as lactic acid, are among the most prominent bioactive compounds supposedly created by LAB activities throughout the fermentation process (Ebringer et al., 2008). The ability to produce each of these metabolites varies among LAB strains. A few LAB strains produce ACE inhibitory peptides, which have antihypertensive characteristics, whilst others produce EPS, which has antidiabetic, cholesterol-lowering, anti-tumor, and immune-modulating activities (Nongonierma and FitzGerald, 2015). Examining LAB strains for the synthesis of EPSs is, therefore, crucial for a variety of reasons. Nakajima et al. reported a comparison of the cholesterol-binding properties of *Lactococcus lactis* with and without EPS production demonstrated by *subsp. cremoris*, the EPS-producing strain had a greater ability to bind cholesterol than the strain not able to produce EPS (Nakajima et al., 1992). In our study, all three isolates produced EPSs (Table 2). The creation of EPSs by LAB considerably contributes to the formation of distinctive qualities in food products such as texture, mouthfeel, and stability (Angelin and Kavitha, 2020; Figure 3).

**Table 2:** Cell surface hydrophobicity (%) and exopolysaccharide production of the LAB isolates.

Isolates	Cell surface hydrophobicity (%) *	Exopolysaccharide production
RAMULAB18	63.32 ± 4.5^b^	+
RAMULAB19	53.70 ± 6.8^a^	+
RAMULAB53	72.81 ± 6.4^c^	+

*Data are provided as mean standard deviation, with the letter “+” representing a positive number. Duncan’s multiple range tests showed that the survival rate averages with a 2 h time interval and superscripts (a–c) differ considerably (*p* ≤ 0.05).

##### Adherence assays

The digestive tract, especially the small intestine, is a dynamic environment, and any bacteria unable to resist the flow by swiftly proliferating or by adhering to intestinal surfaces are washed off by the intestinal transit flow. It is generally accepted that probiotic strains that adhere have a greater chance of colonizing the intestine.

##### Assay for auto and coaggregation

**Figure 4:**
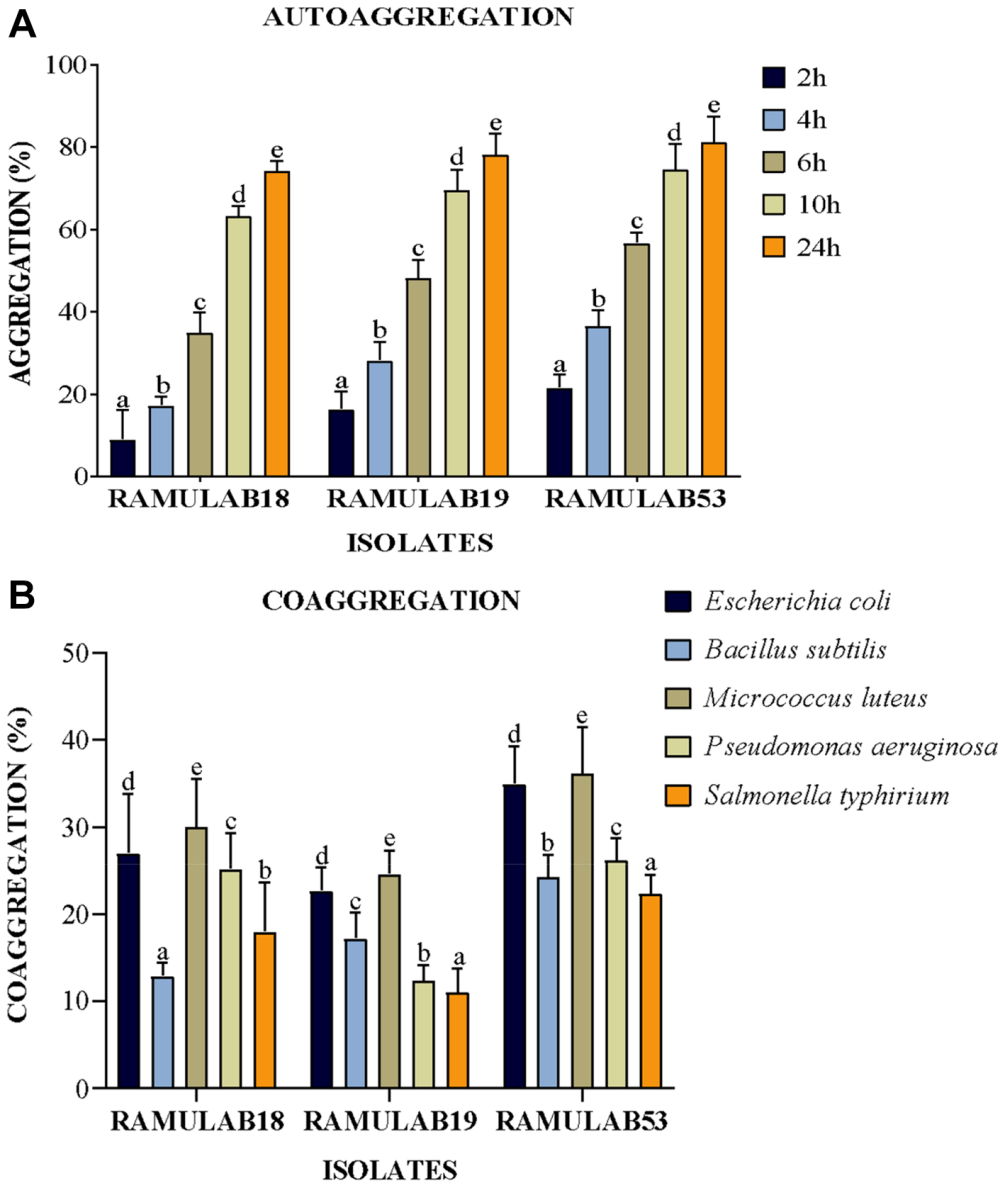
**(A)** Autoaggregation and **(B)** coaggregation of LAB strains at various periods in time and after 2 h of incubation at room temperature. The mean standard deviation depicts the data. Duncan’s multiple range tests showed significant variation (*p* ≤ 0.05) among survival rate averages for the 2 h, as indicated by superscripts (a–e). Cell Surface Hydrophobicity.

The colonization of the organism being treated by probiotic LAB is a beneficial trait; nevertheless, these traits must not only withstand the gastrointestinal host environment but also survive in the GI tract (Gareau et al., 2010). The ability of bacteria to auto-aggregate allows them to cling to the gastrointestinal mucosa, promoting their beneficial effects on the host (Del Re et al., 2000). Given that these same strains can exhibit benefits against pathogens, LAB coaggregation is also viewed favorably. Bacteria that are hydrophobic on their cell surfaces can interact with mucosal cells. The degree of generation of proteins on the cell surface varies between strains of a species, and environmental factors that impact the surface expression of proteins also contribute to variances in cell surface hydrophobicity (Abdulla et al., 2014). A variation in the levels of coaggregation between *Lactobacillus* and pathogens (*L. monocytogenes* and *E. faecalis*, respectively) was observed in the study investigated by Todorov et al. (2007). The investigation by Wang et al. (2018) reported high auto-aggregation qualities, between 65 and 69%, were present in *Pediococcus* strains, in the same study. *Lactobacillus* and *E. faecalis ATCC 29212* co-aggregated at rates of 16–26% and 24–29%, respectively, for *Pediococcus.* The *Weissella* strain had the highest autoaggregation of 79% and the lowest coaggregation of 68% with *E. coli* MTCC 1089 (Elfahri et al., 2016). In our investigation, an exponential increase in the proportion of autoaggregation was reported in all the isolates over time from 2 to 24 h, with RAMULAB53 showing the highest autoaggregation percentage of 81.24% at 24 h. All three isolates reported an autoaggregation activity greater than 74.21% (Figure 4A). All three isolates exhibited significant coaggregation with all five pathogens. Isolates reported the highest coaggregation with *M. luteus* MTCC 1809 ranging from 24.57 to 36.14%, with RAMULAB53 showing the highest coaggregation ability of 36.14% for *M. luteus* (Figure 4B).

The strains under study also displayed substantial favorable characteristics, including cell surface hydrophobicity, as reported in the study by Vidhyasagar and Jeevaratnam (2013) isolate VJ49 exhibited the highest hydrophobicity (49%) than VJ13 (43%) strain (Vidhyasagar and Jeevaratnam, 2013). Caggia et al. (2015) reported strong hydrophobicity in *Pediococcus* (51.3%) and *Lactobacillus* (43–79%) strains. The three isolates in this study had significantly higher levels of autoaggregation >75%, hydrophobicity >53%, and coaggregation which appeared to be strain-specific. This occurs to help maintain the favorable environment of the intestine. In our investigation, the isolates RAMULAB53 and RAMULAB19 reported maximum and minimum hydrophobicity of 72.81 and 53.70%, respectively. The cell surface hydrophobicity was evaluated against xylene (Table 2).

##### Adhesion to HT-29, chicken crop epithelial cells and human buccal epithelial

**Figure 5:**
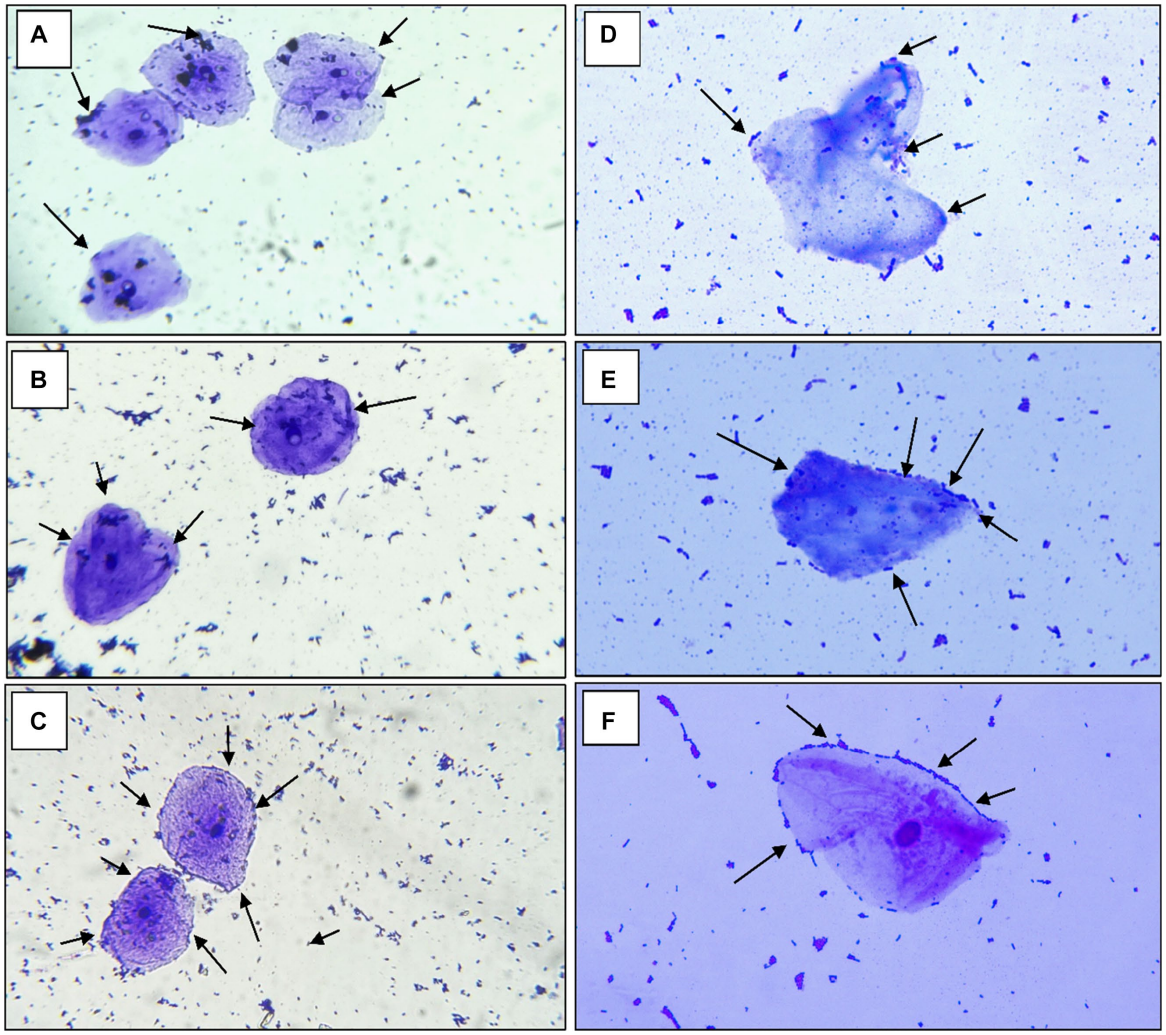
The adhesion of RAMULAB strains to crops of chicken and human buccal epithelial cells is indicated by the adhesion of isolates **(A)** RAMULAB18, **(B)** RAMULAB19, and **(C)** RAMULAB53 to crop epithelial cells of chicken and **(D)** RAMULAB18, **(E)** RAMULAB19, and **(F)** RAMULAB53 to human buccal epithelial cells under a light microscope. As indicated by the black arrow, LAB strains are visible clinging to epithelial cells.

According to the findings of Jaiswal et al. (2022) a strain of *Pediococcus* produces considerable levels of butyrate along with other short-chain fatty acids and has an anti-proliferative effect on colonic cancer cells HT-29 and SW-480 (Joishy et al., 2019). In a study of Alkalbani et al. (2022) the yeasts collected from fermented dairy products and non-dairy products could attach to the HT-29 cell line with an average of 6.3 Log10 CFU/mL after 2 h. According to the research by Hidalgo et al. there were strain-dependent differences in the amount of adhesion to chicken crop epithelial cells: *L. crispatus* CRL 1453 demonstrated the highest levels of adhesion (>19%), while *Lig. sali*var*ius subsp. salivarius* CRL 1417 and *E. faecium* CRL 1385 adhered to a lesser degree (>9 and 2%, respectively) (Huligere et al., 2023). In the study by Lee et al. (2022) reported that *Lactobacillus* spp. Obtained in their study had good adhesion rate toward oral epithelial adhesion. Positive findings emerged from the study, which also looked at LAB adhesion to HT-29 cells and chicken crop and human buccal epithelial cells. The examined LAB strains exhibited a maximum of >84.80% and 35–60 cells/epithelial cell adhesion to HT-29 cell attachment and chicken crop epithelial cells (Figure 5). Aggregation is hence the host’s method of defense mechanism against infection (Chen et al., 2012; Garriga et al., 2015; Lee et al., 2016). In our study strains, RAMULAB53 and RAMULAB18 exhibited the highest and lowest adhesion rates in all three prospects, respectively, as shown in Table 3. The adherence of LAB isolates to HT-29 cells was greater than 64.98%.

**Table 3:** The adhesive capability is determined by calculating the proportion of isolates that bind to HT-29 cells.

Isolates	Cell adherence (%) *
RAMULAB18	64.98 ± 06.57 ^a^
RAMULAB19	71.87 ± 6.80 ^b^
RAMULAB53	84.80 ± 4.74 ^c^

*Data are shown as mean standard deviation. Duncan’s multiple range tests demonstrated that the averages of the survival rate with a 2-h interval of time and superscripts (a–e) differ significantly (*p* ≤ 0.05).

##### Safety assessment

Even though the strains are probiotic and generally recognized as safe (GRAS), FAO/WHO recommendations from 2012 recommend evaluating the safety of putative probiotics using just a restricted set of assays, such as looking at antibiotic resistance patterns (Morelli and Capurso, 2012). The safety and development of the isolated strains were also evaluated in the current investigation. To assess the safety of the LAB, various tests were conducted, including antibiotic susceptibility, DNase activity, hemolytic assay, and antimicrobial assessments. The antibiotic sensitivity of the isolates was investigated to gauge their probiotic potential and to observe the growth of LAB when exposed to antibiotics.

##### Antibiotic sensitivity

Madjirebaye et al. (2022) discovered resistance to tetracycline and cefixime antibiotics in two strains (*L. plantarum* NCU001563 and *S. thermophilus* NCU074001) (Nel et al., 2020). Cortes et al. found no resistance to ampicillin, gentamicin, erythromycin, or tetracycline in strain T40 (*Lacticaseibacillus paracasei*). The isolates were evaluated with thirteen distinct antibiotics in the current study to identify a pattern of resistance to antibiotics. The isolates were assessed against 13 different antibiotics in the study to establish an antibiotic resistance pattern. The results were compared to a reference standards chart. ERY, C, RIF, GEN, AMP, TET, STR, CL, and AZM exhibited sensitivity to all three isolates. Additionally, resistance to VAN, MET, KAN, and CFX was observed (Table 4 and Supplementary Table 1).

**Table 4:** CLSI-based antibiotic susceptibility testing of isolates representing resistance and sensitivity.

Antibiotics	Isolates		
	RAMULAB18	RAMULAB19	RAMULAB53
ERY	S	S	S
C	S	S	S
RIF	S	S	S
GEN	S	S	S
AMP	S	S	S
TET	S	S	S
STR	S	S	S
CL	S	S	S
AZM	S	S	S
MET	R	R	R
KAN	R	R	R
CFX	R	R	R
VAN	R	R	R

S, sensitive; R, resistance.

##### Hemolytic and DNase assay

The DNase enzyme assay was employed to identify bacteria with pathogenic potential that produce the DNase enzyme, responsible for DNA hydrolysis. This confirmed the absence of DNase in the tested isolates, suggesting their suitability for safe use in fermentation and dietary supplements. Subsequent hemolytic testing indicated no hemolysis, further validating the safety of the isolated bacterial strains (Jiang et al., 2018). All three LAB isolates were detected as safe and classed as γ-hemolysis after 48 h of incubation at 37°C with no zone around the colonies (Supplementary Figure 1). DNase activity was another evidence of the probiotic formulation’s safety. No zone was observed for DNase activity, which determines that the isolates without a zone of inhibition were not pathogenic (Supplementary Figure 2). Yadav et al. (2022) discovered that the bacterium *Weissella paramesenteroides* MYPS5.1 isolated from conventional dairy products had neither hemolytic nor DNase activity.

##### Antimicrobial activity

A fundamental aspect of maintaining a healthy microbiota in the digestive tract can be determined by the strains’ capacity to combat infections with antibacterial capabilities. In the current study, all three isolates displayed significant antibacterial activity against the opportunistic pathogens *M. luteus* and *P. aeruginosa*. Jiang et al. reported P.NC8 produced by *L. plantarum* ZJ316 ruptured and permeated the cell membrane in *M. luteus* (Jiang et al., 2018). The study by Ahire et al. reported that antimicrobial activity was detected in the *L. plantarum* UBLP40 isolated from fermented foods against *M. luteus*, *S. aureus*, *P. aeruginosa*, and *E. coli* (Ahire et al., 2021). In our study, the LAB isolates were assessed for antibacterial activity against microbial pathogens. The noteworthy antibiotic activity was shown by the isolates against each of the indicator bacteria. The zone of inhibition is measured on a scale of 6–20 mm (Supplementary Figure 3). All of the isolates exhibited effective antibiotic activity against the opportunistic diseases *M. luteus* and *P. aeruginosa.* A minimal inhibitory action was shown against *B. cereus* and *K. pneumonia* (Table 5). The production of bacteriocin by certain LAB isolates can be the cause of the inhibitory activity.

**Table 5:** The inhibitory activity of *Lactobacillus* strains could be attributed to their ability to generate bacteriocins.

ISOLATES	PATHOGENS									
	*M.*	*P.*	*S.*	*B.*	*E.*	*B.*	*K.*	*S.*	*K.*	*P.*
	*luteus*	*aeruginosa*	*aureus*	*cereus*	*coli*	*subtilis*	*pneumoniae*	*typhimurium*	*aerogenes*	*florescens*
RAMULAB18	+++	+++	++	+	++	++	+	−	−	++
RAMULAB19	+++	++	++	−	+	+	−	+	+	++
RAMULAB53	+++	+++	++	+	++	++	+	+	+	++

Symbols represent zones of inhibition (mm): −, no inhibition; +, weak (<7); ++, moderate (9–15); +++, strong (>15).

##### Molecular characterization and phylogenetic analysis

**Figure 6:**
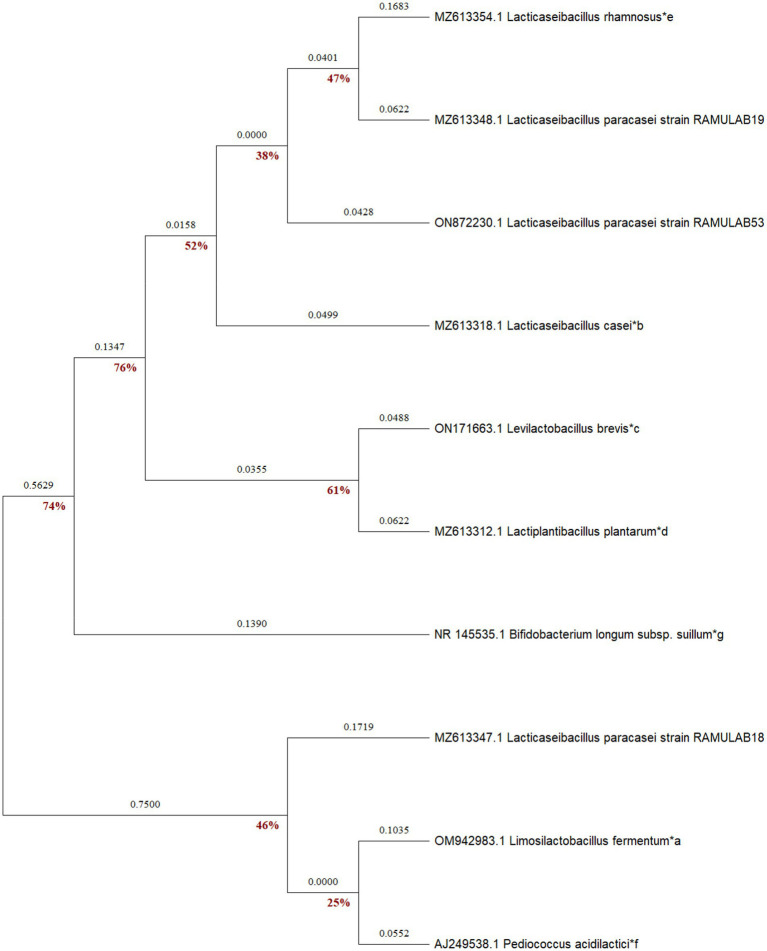
Based on 16s rRNA maximum likelihood bootstrap analysis, the phylogenetic connections of LAB isolates (RAMULAB18, 19, and 53) and reference LAB strains (denoted by *a–e) and outgroup strains (*f and g) were investigated.

The isolates were recognized as LAB based on the sequence data, and phylogenetic analysis using the maximum likelihood bootstrap revealed that the individual strains were *Lacticaseibacillus paracasei* for all three isolates RAMULAB18, RAMULAB19, and RAMULAB53 (Tegopoulos et al., 2021). In our findings, All three isolates were sequenced for the 16S rRNA region and were identified as *Lacticaseibacillus paracasei* (Table 6). MEGA X was used to conduct evolutionary studies of the isolates. This helped in the construction of the phylogenetic tree (Figure 6). It was clear from the phylogenetic grouping that strains with comparable sequences were grouped together and perhaps belonged to the same family. Table 6 lists the NCBI GenBank accession number of the three isolates.

**Table 6:** LAB isolates with GenBank accession numbers have been identified.

Isolates	Sample	Organism	Accession no
RAMULAB18	Milk	*Lacticaseibacillus paracasei*	MZ613347
RAMULAB19	Milk	*Lacticaseibacillus paracasei*	MZ613348
RAMULAB53	Curd	*Lacticaseibacillus paracasei*	ON872230

##### Inhibitory antioxidant assay

**Figure 7:**
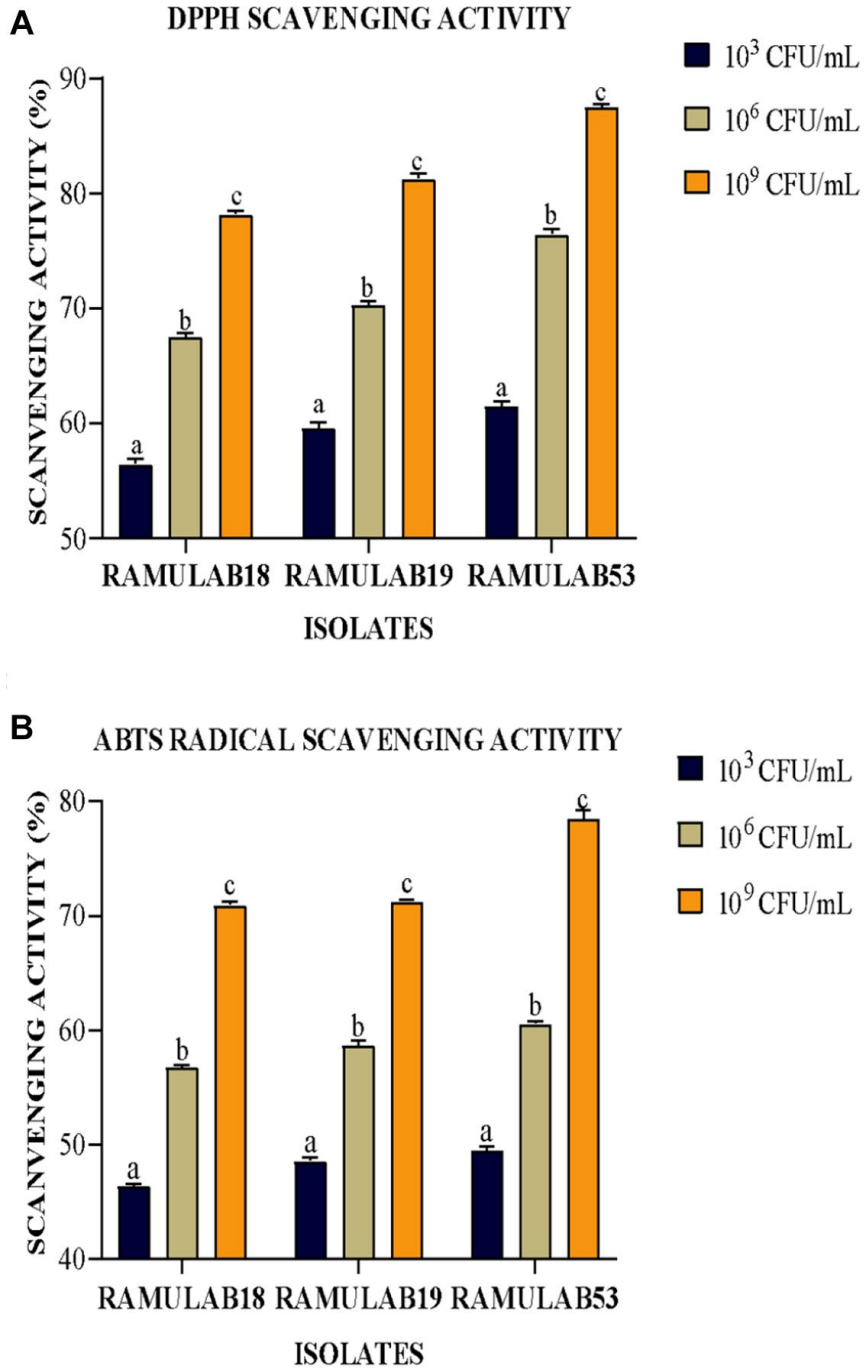
DPPH antioxidant scavenging **(A)** and ABTS radical scavenging **(B)** potential. The results are presented as mean standard deviation. As determined by Duncan’s multiple range test, the means within the same column, denoted by distinct letters (a–c), exhibit significant differences (*p* ≤ 0.05).

Bacterial surface elements are associated with the capacity of intact cells to neutralize free radicals. Among the most harmful reactive oxygen species are hydroxyl and similar radicals, capable of inducing oxidative damage to biomolecules. To convert antioxidants into irrevocably stable compounds, DPPH and ABTS use electrons or hydrogen atoms (Cao et al., 2019). The study by Nongonierma et al. reported: depending on the antioxidant property of the bacterial strain, the proteolytic activity of the *L. rhamnosus* F and *L. reuteri* LR1 strains increased (Nongonierma and FitzGerald, 2015). Oliveira et al. reported in their investigation that the antioxidant activity ranged from 20 to 28% for DPPH inhibition in the intracellular and extracellular contents of *L. satsumensis*, *L. mesenteroides*, and *S. cerevisiae* (de Oliveira Coelho et al., 2019). Our findings align with recent research indicating that intact cells derived from LAB isolates, such as *P. pentosaceus* R1 and *L. brevis* R4, exhibited notably lower ABTS radical scavenging activity compared to cell-free extracts and supernatants (Elfahri et al., 2016). In the present study, the isolates displayed heightened DPPH free radical scavenging activity in correlation with the exponential increase in cell count (CFU/mL) (Figure 7A). RAMULAB53 exhibited the highest radical-scavenging activity (87.45%) at 10^9^ CFU/mL followed by RAMULAB19 and RAMULAB18. The ABTS radicals scavenging activity of the isolates ranged from 46.35 to 49.46% at 10^3^ CFU/mL of cells, RAMULAB53 and RAMULAB18 exhibited the highest and lowest activity, respectively, for all three concentrations of cells (Figure 7B).

##### Inhibitory assay for the carbohydrate hydrolyzing enzymes

**Figure 8:**
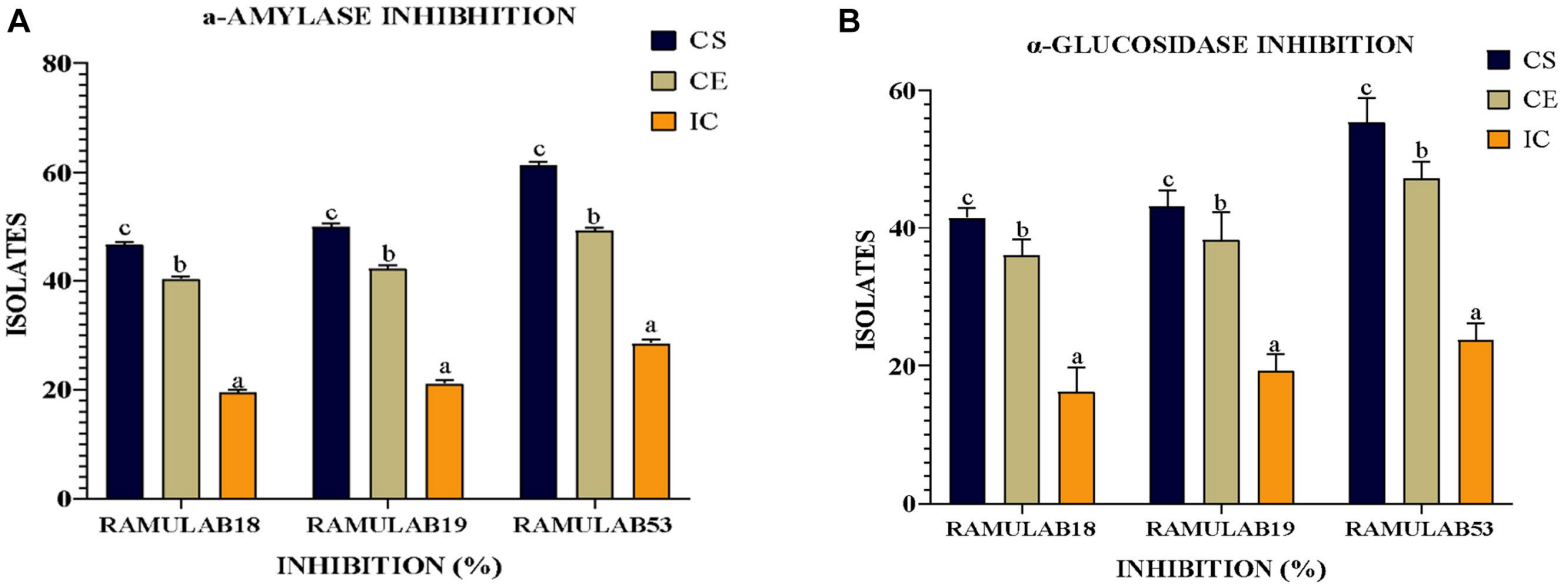
The isolates’ inhibitory activity against **(A)** α-amylase and **(B)** α-glucosidase. The results are presented as mean standard deviation. As indicated by Duncan’s multiple range test, the means within the same column, denoted by distinct letters (a–d), exhibit significant differences (*p* ≤ 0.05).

Measuring α-glucosidase and α-amylase levels allows for the prediction of glucose synthesis inhibition and the gradual lowering of postprandial hyperglycemia. Blood glucose absorption into the small intestine (Ramu et al., 2014; Martiz et al., 2022). Our present research aims primarily to investigate whether probiotic isolates can effectively inhibit the enzymes α-glucosidase and α-amylase, which play a crucial role in glucose metabolism. Based on our data, the RAMULAB isolates’ cell-free supernatants (RL-CS) demonstrated inhibitory potential against α-glucosidase and α-amylase enzymes at rates of 55.45 and 61.31%, respectively. In comparison to commercially available LAB, a study by Son et al. revealed that *L. brevis* KU15006 displayed the highest α-glucosidase inhibitory activity, with values of 24.11% for cell-free supernatant (RL-CS) and 10.56% for cell extract (RL-CE) (Son et al., 2017). The RL-CS and RL-CE had a significantly larger inhibitory impact than the RL-IC in this investigation, demonstrating the presence of inhibitory factors in the cell-free supernatant and extract but the least inhibitory factor in the intact cell. *Lactobacillus* spp. inhibits α-glucosidase and α-amylase. Isolated from food sources demonstrated potential results. These isolates not only improve intestinal health but also lower blood sugar levels. The study by Huligere et al. reported CS, CE, and IC of the isolates had a varying capability of inhibition against α-glucosidase (15.08 to 59.55%) and α-amylase (18.79 to 63.42%) enzymes. In comparison, our investigation also assessed the inhibitory activity of α-amylase and α-glucosidase using the RL-CS, RL-CE, and RL-IC of the isolates. The RL-CS and RL-CE had a notable impact on α-glucosidase and α-amylase for all isolates. The RL-CS, RL-CE, and RL-IC of the isolates inhibited α-amylase to a percentage ranging from 21.15 to 61.31% (Figure 8A), whereas inhibited α-glucosidase to a percentage ranging from 18.24 to 55.45% (Figure 8B). When compared to the supernatant and extract, the intact cells from the isolates showed the least inhibition (Figure 8).

## Conclusion

Diabetes mellitus is a rapidly spreading epidemic with far-reaching social, health, and economic effects. This investigation is an attempt or an alternative to current diabetic management and treatments, as an emerging biotherapy. In this study, *Lacticaseibacillus paracasei* strains isolated from dairy products (milk and curd) revealed significant results with regard to acid-bile-gastrointestinal tolerance, auto and coaggregation capabilities, antibiotic activity, hydrophobicity, and antibacterial properties. CS and CE of isolates have exhibited a significant percentage of inhibition against both carbohydrate hydrolyzing enzymes when compared with the IC of the isolates. In addition, both the CS and CE of the strains have exhibited extensive antioxidant activity by scavenging superoxide anion radicals (DPPH and ABTS). Hence, the three isolates of LAB characterized in this study (RAMULAB18, RAMULAB19, and RAMULAB53) can be considered a viable source for antidiabetic management after further purification. Additionally, *in vivo* studies are needed for further evaluation to bring it out as a supplement.

## Data availability statement

The datasets presented in this study can be found in online repositories. The names of the repository/repositories and accession number(s) can be found in the article/Supplementary material.

## Author contributions

SH: Formal analysis, Investigation, Writing – original draft. CK: Formal analysis, Investigation, Methodology, Writing – original draft. SD: Conceptualization, Data curation, Supervision, Validation, Writing – review & editing. LW: Resources, Software, Writing – review & editing. NF: Resources, Validation, Visualization, Writing – review & editing. RR: Conceptualization, Data curation, Supervision, Validation, Writing – review & editing.
