# Supplementary material for: Investigating the antidiabetic efficacy of dairy-derived Lacticaseibacillus paracasei probiotic strains: modulating α-amylase and α-glucosidase enzyme functions
Source: Front Microbiol. 2023 Dec 4;14:1288487. doi: 10.3389/fmicb.2023.1288487 (PMC10725979; doi:10.3389/fmicb.2023.1288487)
Supplement: Supplementary file 1 [file Data_Sheet_1.docx]

**Investigating the Antidiabetic Efficacy of Dairy-Derived *Lacticaseibacillus paracasei* Probiotic Strains: Modulating α-Amylase and α-Glucosidase Enzyme Functions**

**Sujay S Huligere ^1^, Chandana Kumari V B ^1^, Sudhanva M Desai ^2^, Ling Shing Wong ^3^, Nagma Firdose ^4*^, and Ramith Ramu^1*^**

^1^Department of Biotechnology and Bioinformatics, JSS Academy of Higher Education and Research, Mysore – 570015, Karnataka, INDIA; [sujayhuligere@gmail.com](mailto:sujayhuligere@gmail.com) (S.S.H.); [chandanavb2@gmail.com](mailto:chandanavb2@gmail.com)(C.K.V.B.); [ramith.gowda@gmail.com](mailto:ramith.gowda@gmail.com) (R.R.)

^2^Department of Chemical Engineering, Dayanand Sagar College of Engineering, Bengaluru – 560078, Karnataka, INDIA; [desai-chml@dayanandasagar.edu](mailto:desai-chml@dayanandasagar.edu) (S.M.D.)

^3^ Faculty of Health and Life Sciences, INTI International University, Nilai, Malaysia; [lingshing.wong@newinti.edu.my](mailto:lingshing.wong@newinti.edu.my) (L.S.W.)

^4^ Department of Pharmacology, JSS Medical College, JSS Academy of Higher Education and Research, Mysore – 570015, Karnataka, INDIA; [nagma@jssuni.edu.in](mailto:nagma@jssuni.edu.in) (N.F.)

* Correspondence: [nagma@jssuni.edu.in](mailto:nagma@jssuni.edu.in); [ramith.gowda@gmail.com](mailto:ramith.gowda@gmail.com)

**Supplementary Material**

**Table 1.** Breakpoints of sensitivity/resistance (S/R) in the mm inhibitory zone of respective antibiotics based on CLSI, 2018.

| **Sl.No.** | **Antibiotic** | **the inhibitory zone(S/R mm)** |
| --- | --- | --- |
| 1 | Chloramphenicol (C) | (≥18/≤12) |
| 2 | Gentamicin (GEN) | (≥15/≤12) |
| 3 | Clindamycin (CD) | (≥19/≤14) |
| 4 | Ampicillin (AMP) | (≥17/≤14) |
| 5 | Kanamycin (K) | (≥18/≤12) |
| 6 | Tetracycline (TET) | (≥19/≤14) |
| 7 | Vancomycin (V) | (≥17/≤14) |
| 8 | Erythromycin (E) | (≥23/≤13) |
| 9 | Streptomycin (STR) | (≥15/≤12) |
| 10 | Rifampicin (RIF) | (≥20/≤16) |
| 11 | Methicillin (MET) | (≥22/≤4) |
| 12 | Azithromycin (AZM) | (≥13/≤12) |

**
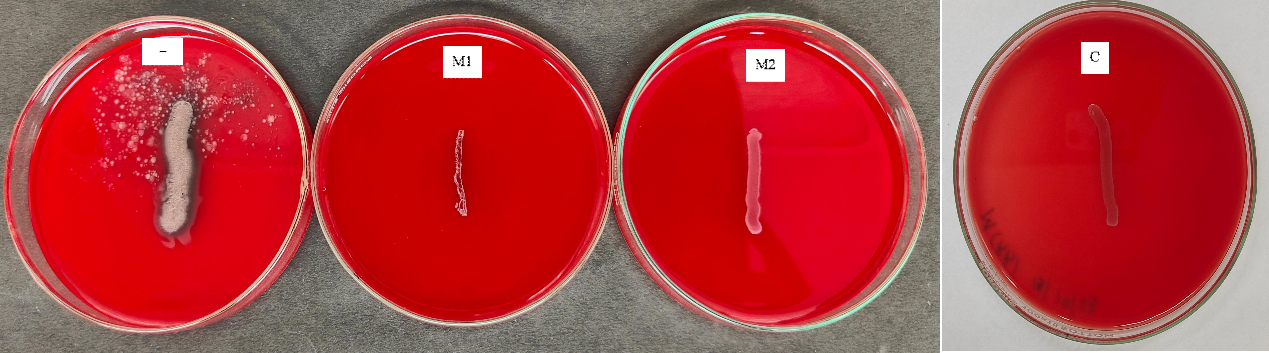
Figure 1.** Haemolytic activity. The Plates (-) negative control of *S. aureus* shows α-haemolysis and RAMULAB isolates showed γ-haemolysis (M1) RAMULAB18, (M2) RAMULAB19, and (C) RAMULAB53.

**
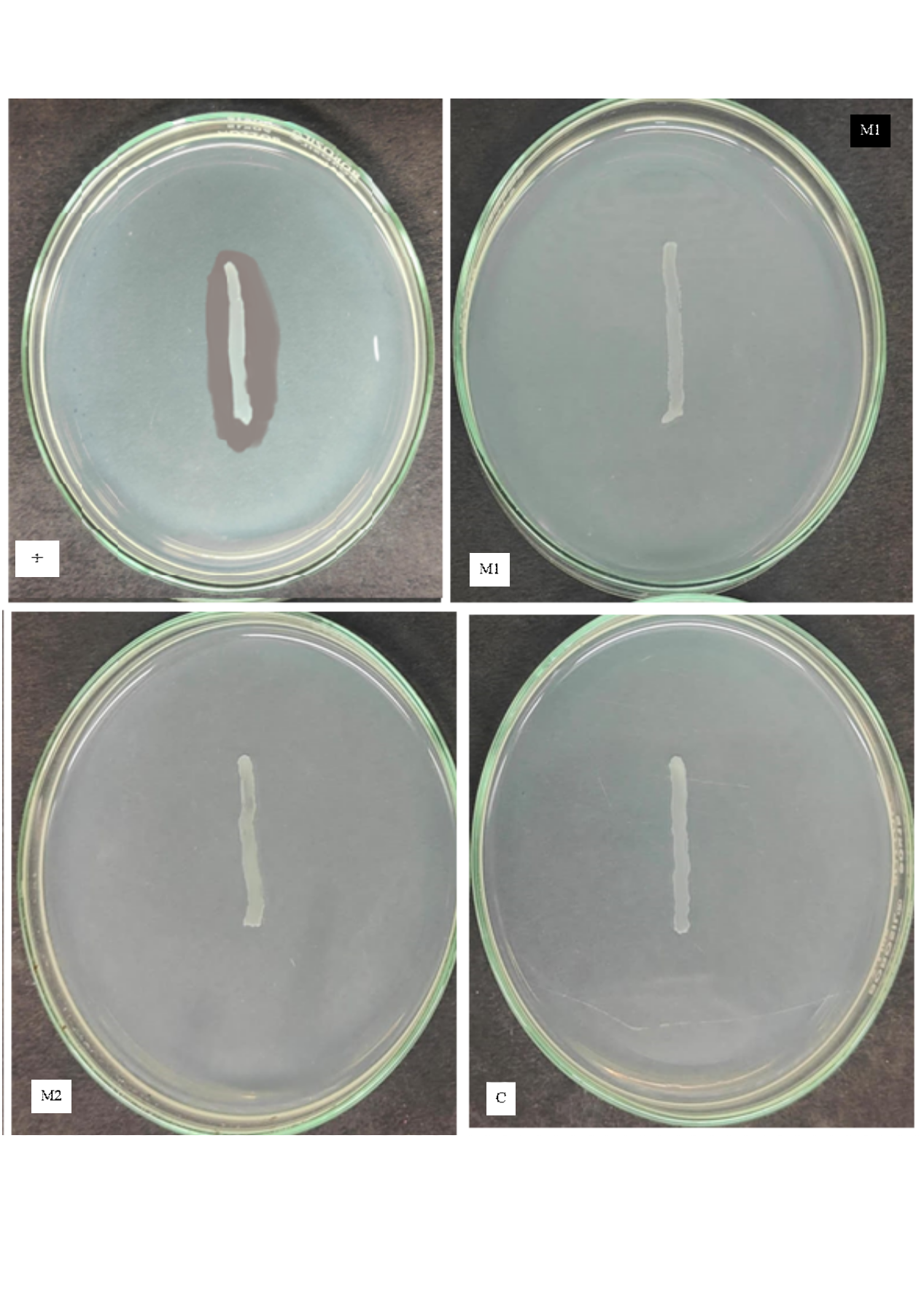
**

**Figure 2.** DNase activity. The Plates (+) Positive control of *S. aureus* shows zone formation and RAMULAB isolates showed no zone (M1) RAMULAB18, (M2) RAMULAB19, and (C) RAMULAB53.

**
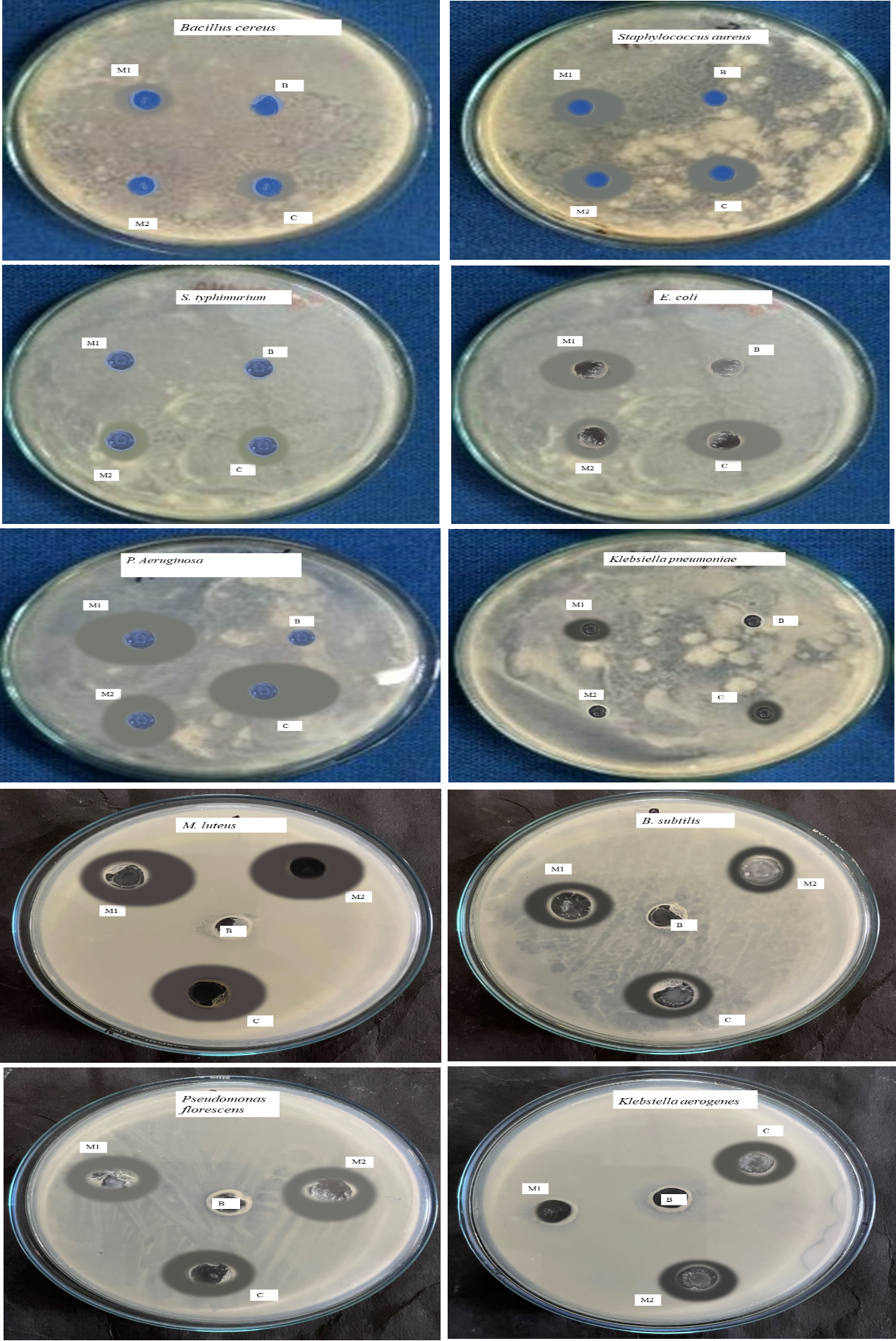
**

**Figure 3.** Antibacterial activity of RAMULAB isolates: (M1) RAMULAB18, (M2) RAMULAB19, and (C) RAMULAB53 against the pathogen by well diffusion method.
